# Cutaneous Lesions Anticipating Accelerated Phase of Multidrug Resistant Chronic Myeloid Leukemia Responsive to Ponatinib

**DOI:** 10.4084/MJHID.2016.016

**Published:** 2016-02-20

**Authors:** Massimo Breccia, Gioia Colafigli, Luisa Quattrocchi, Elisabetta Abruzzese, Giuliana Alimena

**Affiliations:** 1 Department of Cellular Biotechnologies and Hematology, Sapienza University, Rome, Italy; 2 Hematology, S. Eugenio Hospital, Rome, Italy

**Dear Editor,**

Ponatinib (formerly known as AP24534, Ariad Pharmaceuticals, Cambridge, MA) is the first tyrosine kinase inhibitor (TKI) to have potent and consistent activity against BCR-ABL1 with the T315I mutation.^1^ The drug was developed based on a scaffold that, unlike current available TKI, does not make a hydrogen bond with T315, and has a long and flexible ethynil tri-carbon linker which permits its accommodation in the catalytic domain even in the presence of the bulky side chain of isoleucine at residue 315.^2^ Results from a recently reported phase I study of Ponatinib in patients with advanced hematological malignancies showed that among 38 patients with chronic phase CML (CP-CML) enrolled, a complete hematologic response (CHR) was obtained in 95% and a complete cytogenetic response (CCyR) in 53%.^3^ To confirm what recently reported,^4^ we describe the activity of ponatinib as fourth line in a CML patient in accelerated phase (AP) resistant to prior TKIs who had cutaneous manifestation and achieved improvement of the skin lesions and of disease. A 43-year old male was diagnosed as having CP-CML, with intermediate Sokal risk, in February 2006. After a short period of cytoreduction with hydroxyurea, he was started on standard dose imatinib and achieved a CHR after 2 weeks, but only reached a minor CyR at 3 months and a partial CyR at 6 months. At that time mutational test was negative: patient admitted sporadic scarce adherence and blood level testing repeated at two different times revealed imatinib in the considered normal range (> 1100 mg/dl). Dasatinib 100 mg/day was started because the patient was considered as a failure: after 3 months CCyR was achieved and molecular analysis performed at that time showed a consistent but not optimal response (BCR-ABL ratio 2.9% IS). Patient continued with the drug and maintained CCyR but, after achieving MMR at 12 months, did never reach a stable deep molecular response. After 38 months, he suddenly lost hematologic response while admitting the discontinuation of the drug for a long period. At that time a CBA displayed a cytogenetic clonal evolution (47, XY, t(9;22) (q34;q11), i(17)(q10)[4]/46, XY [17]) and molecular analysis revealed a significant increase of BCR-ABL ratio (53%). Mutational analysis repeated at that time did not show the presence of mutations. While a MUD research was activated, Nilotinib at the dose of 400 mg BID was started. The patient reached a CHR after 2 weeks and a CCyR after 3 months but again he did never reach a stable molecular response (BCR-ABL ratio of 1.2% after 6 months). After 5 months of treatment, he developed diffuse scaly patches of red and white hue that were initially diagnosed as plaque psoriasis (figure 1). Only local therapy was prescribed, but an inflammation and exfoliation of the skin over most of the body surface was soon observed, concomitantly with appearance of thrombocytopenia, anemia, and leucocytosis. At that time we believed that skin lesions were associated to progression of disease; a bone marrow analysis showed a disease progression to accelerated phase: CBA identified a karyotype mosaicism (46, XY, t(9;22) (q34;q11) [2]/ 46, XY, t(9;22), i(17)(q10) [3]/ 45,XY,t(9;22), i(17)(q10), -Y [4]/ 46, XY [12]), and mutational test revealed the emergence of a L387F mutation. Nilotinib was discontinued and, due to severe leukocytosis, patient was started on low dose chemotherapy (vindesine 5 mg total dose). Compassionate use ponatinib was then initiated at the dose of 45 mg/day. After 1 week, patient reached a CHR with persistence of anemia and after 2 weeks showed an improvement of skin lesions that completely disappeared after 4 weeks of treatment (figure 2). After 5 weeks, patient developed a bronchopneumonia that required discontinuation of the drug and antibiotic treatment: during the discontinuation of Ponatinib for 3 weeks, the skin lesions reappeared. When the drug was restarted at the same dose, the skin lesions disappeared again after two weeks, indirectly suggesting that skin lesions are direct manifestation of disease. After achieving CHR and PCyR, patient progressed and finally died for sepsis. In a phase I trial with Ponatinib, 74 patients (64 with refractory CML or Ph+-ALL) were recruited. Patients received the drug at doses ranging from 2 to 60 mg once daily. The most common side effects were thrombocytopenia (23%), rash (22%) and arthralgia (15%).^4^ Recently, the efficacy and safety of ponatinib were evaluated in a phase II trial, named PACE:^5^ of 449 patients enrolled, 270 were in CP and the majority had previously received two or more lines of therapy. After a median follow-up of 9 months, overall 56% of patients achieved the primary endpoint (MCyR) in a median time of 2.8 months, with 51% of patients being previously resistant to dasatinib and/or nilotinib and 70% of patients having a T315I. A CCyR was achieved in 46% of cases, MMR in 34% and MR4.5 in 15%. Ninety-one percent of patients maintained MCyR at 12 months. The results of the trial also proved that Ponatinib inhibits both wild-type (IC50=0.37 nM) and T315I mutated (IC50=2.0 nM) BCR-ABL1, while having activity against several common BCR-ABL1 mutations such as E255K, Y253H and G250E. The most important adverse events reported were abdominal pain, skin rash and increase of amylase with pancreatitis, which occurred in 7% of patients. As shown also in present case, skin lesions should be considered as a possible sign of progression and ponatinib can be safely used, also in patients in advanced CML phase associated with extramedullary manifestations of disease. Further studies in patients with extramedullary localizations are warranted and in particular in patients with skin localization to understand if ponatinib is able to overcome this possible sanctuary of disease.

## Figures and Tables

**Figure 1:**
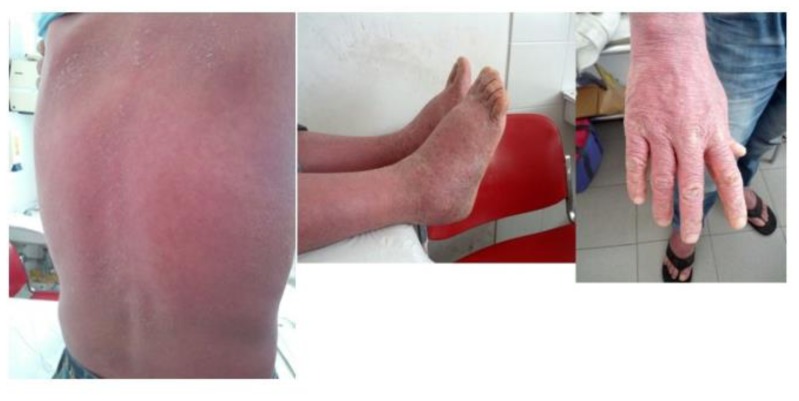
Maculo-papular and erythematous skin lesions before ponatinib treatment

**Figure 2:**
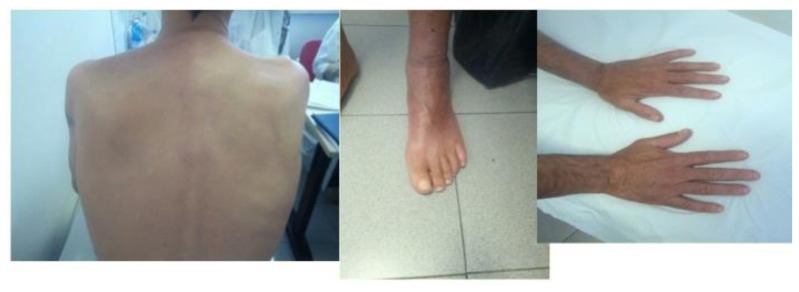
Resolution of skin lesions after ponatinib treatment
